# Combination therapy with antioxidants improves total motile sperm counts: A Preliminary Study

**DOI:** 10.1002/rmb2.12308

**Published:** 2019-11-28

**Authors:** Kazutaka Terai, Shigeo Horie, Shinichiro Fukuhara, Yasushi Miyagawa, Kazuhiro Kobayashi, Akira Tsujimura

**Affiliations:** ^1^ Department of Urology Juntendo University Graduate School of Medicine Tokyo Japan; ^2^ Men's Health Clinic Tokyo Tokyo Japan; ^3^ Department of Urology Osaka University Graduate School of Medicine Osaka Japan; ^4^ Department of Urology Juntendo University Urayasu Hospital Chiba Japan

**Keywords:** antioxidant, hochu‐ekki‐to, male infertility, oligoasthenozoospermia, total motile sperm count

## Abstract

**Purpose:**

Optimal strategies to treat idiopathic male infertility have remained unclear. The aim of this study was to evaluate the effectiveness of combination antioxidant therapy with several vitamins and supplements on semen parameters.

**Methods:**

Thirty‐one men with oligozoospermia and/or asthenozoospermia evaluated by a Makler counting chamber were randomly assigned to two treatment groups: a combination of antioxidant supplements (L‐carnitine, zinc, astaxanthin, coenzyme Q_10_, vitamin C, vitamin B_12_, and vitamin E) and a Chinese herbal medicine, hochu‐ekki‐to (HE). Serum endocrinological profiles and semen parameters, especially total motile sperm count, were compared between before and after 12 weeks of treatment in both groups.

**Results:**

In the supplement group, endocrinological findings were not significantly improved. The semen parameters of semen volume, sperm concentration, and sperm motility were not statistically significantly improved, whereas total motile sperm count was significantly improved. In contrast, none of the endocrinological factors or semen findings were significantly improved by the Chinese herbal medicine although semen concentration, semen motility, and total motile sperm count showed a tendency to increase.

**Conclusion:**

Because combination antioxidant therapy could improve sperm motility significantly for patients with idiopathic oligoasthenozoospermia, our supplement could be one treatment option for idiopathic male infertility.

## 1 INTRODUCTION

In the current situation of aging societies with falling birth rates and shrinking populations, infertility may be a national problem in many developed countries. If men attempt sexual intercourse as usual on the day of ovulation but they are not blessed with children after more than 1 year of trying, they are categorized as being infertile. Although infertility had been accepted as a female issue by the public for a long time, it is now being publicized that half of the causes of infertility are due to male factors. Recently, the concept of male infertility has also been recognized as an important issue in the field of reproductive medicine. Furthermore, meta‐regression analyses including 185 studies regarding semen quality recently showed shocking data that the sperm concentrations and sperm counts in the study patients had decreased by 52.4% and 59.3% from 1973 to 2011, respectively.^1^ Thus, as this fact becomes widely known, semen quality is gaining increased attention in the public. In Japan, a nationwide survey of male infertility in 2017 indicated that the major etiology of infertility was failure of spermatogenesis (82.6%).^2^ Furthermore, about a half of the patients were classified into the group with idiopathic male infertility for an unknown reason, that is, idiopathic failure of spermatogenesis.

Because optimal strategies for treating idiopathic male infertility have remained unclear, the adequate strategy of treatment for such patients with idiopathic failure of spermatogenesis is attracting the most attention now among several clinical issues regarding male infertility. It was previously reported in a survey of 7745 practicing American Urological Association members from the United States that empirical medical therapy is used by two‐thirds of survey respondents for idiopathic male infertility.^3^ Furthermore, it was emphasized in the guideline for male infertility by the European Urological Association that there is little scientific evidence for an empirical approach although a wide variety of empirical drug treatments for idiopathic male infertility have been used. However, in the same guideline, it was also stated that men taking oral antioxidants had an associated significant increase in sperm parameters.^4^

For a long time now, seminal oxidative stress, which results from an imbalance between the production of reactive oxygen species (ROS) and the scavenging by seminal antioxidants, has been believed to be one of the main factors in the pathogenesis of sperm dysfunction in male infertility. Furthermore, several investigators have shown that seminal antioxidant capacity is suppressed in infertile men with high ROS levels.^5^, ^6^ These findings should be evidence to encourage clinicians to treat infertile men with antioxidant supplements, although scientifically acceptable evidence for these pharmacological treatments is limited. Generally, idiopathic male infertility is caused by several problems including endocrinological disorder, testicular failure due to varicocele and/or vascular insufficiency, lifestyle factors such as smoking, obesity, sleep deprivation, and metabolic syndrome, and genetic factors. Thus, it is highly unlikely that a single drug will be effective for all patients with idiopathic male infertility in light of the wide variety of causes, even though the drug is a vitamin or supplement with antioxidant effect. In Japan also, empirical medical therapy with several herbal medicines, vitamins, and supplements with antioxidant effect has been undertaken reluctantly. However, a nationwide survey reported that the efficacy was no more than 23.7% (207/872 cases) for herbal medicine, 18.0% (283/1571 cases) for vitamins and 41.3% (135/327 cases) for supplements.^2^

On the basis of these situations, we chose several vitamins and supplements with antioxidant effect after reviewing previous studies regarding the efficacy of antioxidants on semen parameters and administered combination antioxidant therapy to patients with idiopathic male infertility. The aim of this study was to evaluate the effectiveness of our combination treatment on semen parameters, especially that of total motile sperm count.

## 2 PATIENTS AND METHODS

### 2.1 Patients

From August 2015 to March 2017, we enrolled 31 infertile male patients with oligozoospermia and/or asthenozoospermia who visited at Department of Urology, Juntendo University Graduate School of Medicine or Men's Health Clinic Tokyo and agreed to participate in our study. Inclusion criteria were participant aged between 20 and 60 years old and the presence of abnormal semen parameters indicating oligozoospermia and/or asthenozoospermia. Semen samples were obtained by masturbation, and all samples were examined within 1 hour of ejaculation. A semen test was performed after 4 or more days of abstinence by use of a Makler semen counting chamber. Oligozoospermia was defined as a sperm concentration of <15 million/mL, and asthenozoospermia was defined as sperm motility of <40% according to the 2010 World Health Organization criteria. Patients with azoospermia, severe oligozoospermia (sperm concentration <5 million/mL), and severe asthenozoospermia (sperm motility <5%) were excluded. Conversely, patients with a total motile sperm count of more than 30 million were also excluded. Patients with medical (varicocele or cryptorchidism) or surgical conditions that can result in infertility, a history of cancer chemotherapy, drug or other substance abuse, administration of androgens, anti‐androgens, and immunosuppressants, severe kidney disease (serum creatinine >2.0 mg/dL) and liver insufficiency (serum bilirubin >2.0 mg/dL), and endocrinopathy were also excluded.

### 2.2 Methods

Several factors including age, body mass index, smoking habit, and bilateral testicular volumes evaluated by orchidometer were assessed. Presence of a varicocele was determined by ultrasonography of the scrotum with the Valsalva maneuver. The serum levels of luteinizing hormone (LH), follicle‐stimulating hormone (FSH), and total testosterone were measured by radioimmunoassay. Based on previous reports, our antioxidant supplement for male infertility includes L‐carnitine, zinc, astaxanthin, coenzyme Q_10_, vitamin C, vitamin B12, and vitamin E (Table 1). The patients with idiopathic male infertility were randomly assigned to the two groups in a random sequence. Random assignment was performed by the central registration system of an independent organization. For randomization, age, testicular volume, serum FSH concentration, sperm concentration, and sperm motility were used as factors in the assignment. Patients took either the antioxidant supplement three times a day (n = 15; supplement group) or the hochu‐ekki‐to (HE): a Chinese herbal medicine, three times a day (n = 16; HE group) over a 12‐week period. All semen tests were performed at the pretreatment baseline and at 12 weeks. Serum concentrations of LH, FSH, and testosterone were compared between before and after each treatment. Semen parameters such as semen volume, sperm concentration, sperm motility, and total motile sperm count were also compared between before and after each treatment.

**Table 1:** Antioxidant supplements

Component	Dose
L‐Carnitine	750.1 mg
Zinc	30 mg
Astaxanthin	16.05 mg
Coenzyme Q10	90.26 mg
Vitamin C	1000 mg
Vitamin B12	60.1 μg
Vitamin E	150 mg

We explained the aim and method of the study to all patients. All patients were aware of and accepted that they would receive antioxidant supplements or HE. All patients signed an informed written consent form before entering the study, and they were informed that they could terminate their cooperation with us whenever they wanted without any consequences. This study was approved by the institutional review board of Juntendo University School of Medicine and Men's Health Clinic Tokyo.

The data are presented as mean values ± standard deviation, and comparisons of patient characteristics were statistically evaluated by Mann‐Whitney *U* test and chi‐square analysis. The changes of endocrinological findings and semen parameters were investigated by Paired Student t test. A *P‐*value < 0.05 was considered to indicate statistical significance. Statistical analysis was performed using JMP^®^ 13 (SAS Institute Inc).

## 3 RESULTS

**Figure 1:**
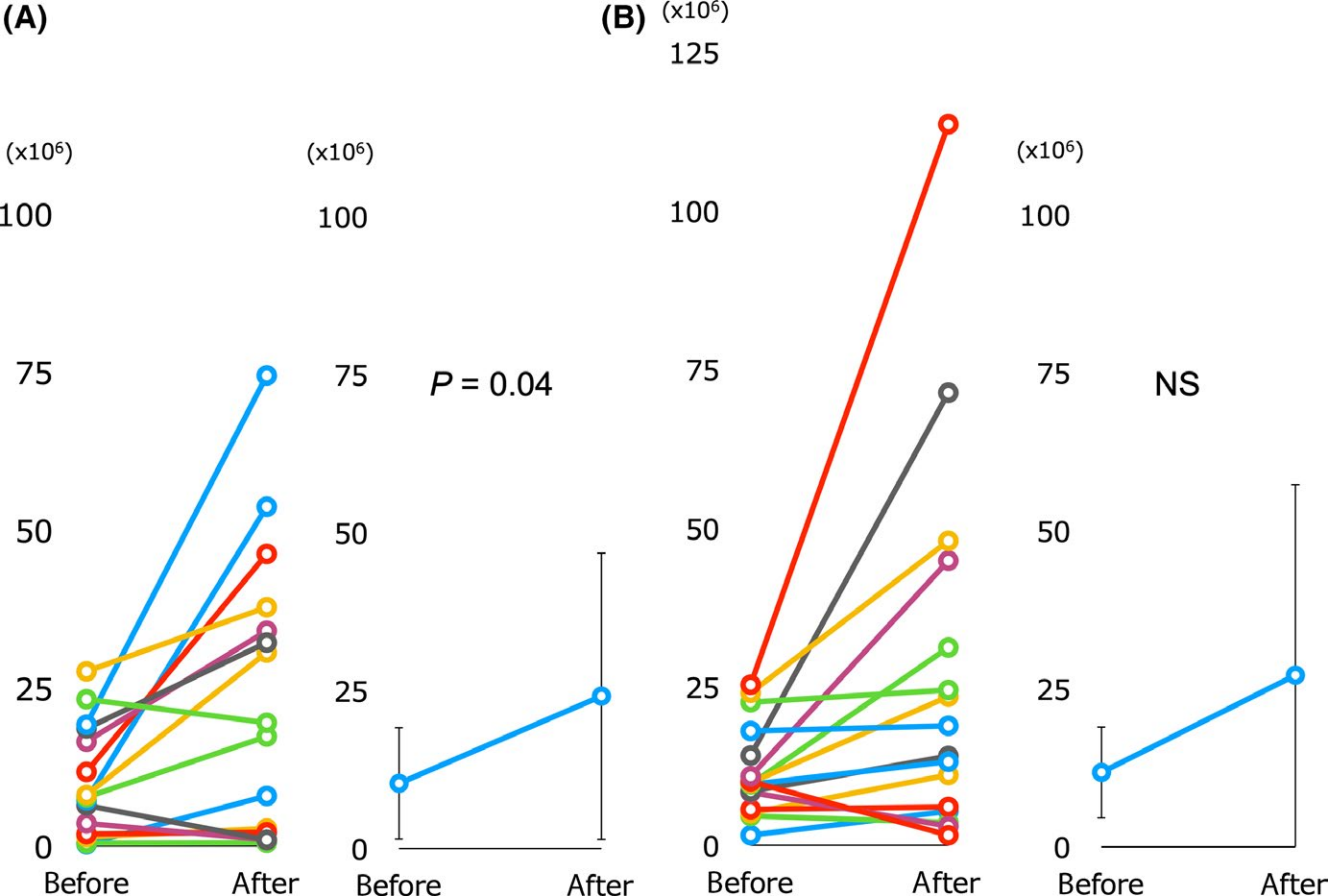
Changes in the total motile sperm count. A, Supplement group, B, HE (hochu‐ekki‐to) group. NS, not significant

Patient characteristics are shown in Table 2. Neither age, testicular volume, and endocrinological findings nor semen parameters including semen volume, sperm concentration, sperm motility, and total motile sperm count were statistically significantly different between the groups at the baseline measurement. The rates of smokers and varicoceles (grade 1) were almost the same between the two groups (Table 2). Endocrinological findings including LH, FSH, and testosterone were not significantly improved in the supplement group. Although the semen parameters of semen volume, sperm concentration, and sperm motility were not improved statistically, total motile sperm count was significantly improved (10.3 ± 8.5 × 10^6^ vs 24.1 ± 21.9 × 10^6^, *P* = .04; Table 3 and Figure 1). In contrast, none of the factors related to both endocrinological and semen findings were significantly improved in the HE group, although semen concentration, semen motility, and total motile sperm count showed a tendency to increase.

**Table 2:** Patient characteristics

Characteristic	Supplement	HE	*P*
Number of cases	15	16	–
Age	39.4 ± 6.8	3.8 ± 5.8	.78
Right testicular volume (mL)	19 ± 3.3	18 ± 3.3	.15
Left testicular volume (mL)	18 ± 4.6	17 ± 4.1	.29
LH (mIU/mL)	4.1 ± 2.6	3.9 ± 2.1	.99
FSH (mIU/mL)	6.3 ± 4.3	5.8 ± 3.0	.62
Total testosterone (ng/mL)	5.4 ± 2.3	4.8 ± 0.9	.06
Semen volume (mL)	3.0 ± 1.9	2.8 ± 1.5	.84
Sperm concentration (×10^6^/mL)	37.7 ± 46.4	24.6 ± 28.1	.36
Sperm motility (%)	21.4 ± 16.1	30.2 ± 15.4	.14
Smoker	3 (20.0%)	3 (18.8%)	.92
Varicocele (G1)	3 (20.0%)	5 (31.3%)	.23

Abbreviations: FSH, follicle‐stimulating hormone; HE, Hochu‐ekki‐to; LH, luteinizing hormone.

**Table 3:** Endocrinological findings and semen parameters before and after the treatment

Number of cases	Supplement			HE		
	15			16		
	Before	After	*P*	Before	After	*P*
LH (mIU/mL)	4.1 ± 2.6	3.8 ± 1.3	.70	3.9 ± 2.1	4.0 ± 1.8	.83
FSH (mIU/mL)	6.3 ± 4.3	5.4 ± 2.7	.53	5.8 ± 3.0	6.3 ± 3.4	.67
Total testosterone (ng/mL)	5.4 ± 2.3	4.6 ± 1.0	.26	4.8 ± 0.9	4.4 ± 1.0	.29
Semen volume (mL)	3.0 ± 1.9	2.7 ± 1.6	.70	2.8 ± 1.5	2.8 ± 1.2	.93
Sperm concentration (×10^6^/mL)	37.7 ± 46.4	49.1 ± 37.7	.68	24.6 ± 28.1	35.2 ± 35.4	.37
Sperm motility (%)	21.4 ± 16.1	31.4 ± 18.3	.33	30.2 ± 15.4	34.0 ± 17.6	.54
Total motile sperm count (×10^6^)	10.3 ± 8.5	24.1 ± 21.9	.04	11.7 ± 6.9	27.1 ± 29.1	.06

Abbreviations: FSH, follicle‐stimulating hormone; HE, Hochu‐ekki‐to; LH, luteinizing hormone.

## 4 DISCUSSION

As developed society has been heading toward lower birth rates and increased aging, infertility has gained more and more attention. Especially, the concept of male infertility has attracted a great deal of public attention. Many infertile men have abnormal semen analyses for which the etiology is often poorly explained. Among the types of male infertility, the most often and most troublesome one is idiopathic, indicating the apparent lack of a cause for the worsened semen quality. Etiologies of idiopathic male infertility can include environmental, dietary, medical, genetic, and physiologic factors. Because it is speculated that a broad range of causes may be associated with idiopathic failure of spermatogenesis, a uniform treatment strategy is not realistic. In Japan and other countries, the fact is that empirical medical therapy depends on the preference of each physician. In this circumstance, HE is a Chinese herbal medicine that has been used for unexplained male infertility with some favorable clinical results. HE may promote the synthesis of several proteins that might be related to the functional maturation of spermatozoa in the epididymis.^7^, ^8^ In the present study, we attempted to find a more favorable supplement for idiopathic male infertility, that is, unexplained spermatogenetic failure.

ROS have been implicated as a potential cause of male infertility. Several clinical studies have shown that elevated concentrations of ROS are often found in patients with infertility.^9^, ^10^ Oxidative stress in the genitourinary system can damage sperm, resulting in decreased motility, lipid peroxidation, increased DNA damage, and decreased oocyte‐sperm fusion.^11^ Furthermore, it was also reported that ROS have a significant impact on spermatogenesis and on sperm function. Indeed, statistically significant negative correlations between oxidation reduction potential and normal sperm morphology, progressive motility, and sperm concentration have been reported.^12^ The seminal plasma contains natural antioxidants such as vitamins C and E, superoxide dismutase, glutathione, uric acid, and the polyamine spermine, which acts as a free radical scavenger.^13^ These findings may be reasons why the guideline for male infertility by the European Urological Association stated that men taking oral antioxidants had an associated significant increase in sperm parameters. L‐carnitine (beta‐hydroxy‐gamma‐N‐trimethylamino‐butyric acid) is a water‐soluble vitamin‐like amino acid, which is associated with the preservation of membrane integrity, mitochondrial function, and inhibition of apoptosis. Because sperm motility is initiated with the simultaneous increase in the concentration of free L‐carnitine in the epididymal lumen,^14^ it has been expected that dosing with L‐carnitine can improve sperm motility in patients with male infertility. Indeed, a double‐blind crossover trial with placebo clearly showed a statistically significantly greater improvement in semen quality after the L‐carnitine therapy for sperm concentration and total and forward sperm motility than after the placebo cycle.^15^

Zinc is also expected to be important for reproduction because it serves as a cofactor for more than 80 metallic enzymes involved in cellular development processes. Because the concentration of zinc is very high in the seminal fluid compared with blood, it is speculated to be involved in the sperm's oxidative metabolism. Furthermore, concentrations of zinc in seminal plasma were closely associated with sperm concentration and motility.^16^ Indeed, it was already reported that the volume of semen, progressive sperm motility, and total normal sperm count were increased after zinc supplementation.^17^

Astaxanthin is a carotenoid extracted from the algae *Haematococcus pluvialis*. Because its high potency as an antioxidant is well known, it is expected to improve semen quality. A double‐blind randomized trial studying sperm motility showed a trend toward improvement during treatment with astaxanthin, although the changes did not reach the level of significance.^18^ Coenzyme Q_10_, which has gained increased attention in the public health sector, appears to play an important role as a liposoluble antioxidant for cell membranes and lipoproteins.^19^ Recently, a double‐blind randomized trial with coenzyme Q_10_ and placebo for patients with idiopathic oligoasthenoteratospermia revealed significant improvement in sperm density and motility with coenzyme Q_10_ therapy.^20^, ^21^

Among the vitamins, vitamin C is a representative antioxidant that is freely available in citrus fruits and fresh berries. A study of infertile patients with oligozoospermia showed that the mean sperm count was increased after 2 months of vitamin C intake, and mean sperm motility was also increased significantly.^22^ Recently, a randomized trial of patients undergoing varicocelectomy also showed a statistically significant improvement in sperm motility in the vitamin C treatment group compared with the placebo group.^23^ Vitamin B12 has been used mostly in the clinical setting of male infertility in Japan as an antioxidant. Basically, it is chosen empirically because of its well‐known antioxidant potential. However, there is certainly some evidence for the efficacy of vitamin B12 on semen parameters, particularly sperm count, in patients with male infertility.^24^ Recently, another study revealed the efficacy of vitamin B12 on semen quality and sperm physiology.^25^ Regarding vitamin E, it is also known that vitamin E inhibits the production of ROS in infertile males. A randomized control study of patients with idiopathic oligoasthenozoospermia was conducted for 6 months in three groups: vitamin E alone, clomiphene citrate alone, and a combination of both drugs. The results showed a significant improvement in mean total sperm motility in the group receiving clomiphene citrate alone although the improvement was most apparent in the combination therapy group.^26^ Furthermore, another combination therapy with selenium and vitamin E administered to 690 men with oligoasthenozoospermia showed a significant improvement in sperm motility, morphology, or both.^27^ Moreover, spontaneous pregnancy occurred in 10.8% of cases in the treatment group.

On the basis of these previous reports, we manufactured a specific combined supplement for male infertility consisting of L‐carnitine, zinc, astaxanthin, coenzyme Q_10_, and vitamins C, B12, and E. Our combination supplement significantly improved the total motile sperm count over a 3‐month period, whereas the improvement by HE did not reach statistical significance. Although it is well known that HE also plays a role in superoxide suppression and antioxidant capacity, total motile sperm count was not improved even by it. Thus, we speculated that our supplements causing enhanced antioxidant capacity may have potentials for the treatment for idiopathic male infertility.

However, the present study has several limitations. First, the number of patients included in the study was small to induce the evidential conclusion. Second, the results of semen testing using the Makler semen counting chamber are not accurate for measurements of sperm concentration and sperm motility rate.^28^ We understand that the semen test recommended by the World Health Organization is a reliable method, but it is complicated and takes time to obtain test results. Third, the test was conducted only once. It is well known that semen findings change depending on physical conditions. Because the present study was performed during routine clinical duty, we did not have the choice to use an evaluation method other than the Makler semen counting chamber. Although a large‐scale study using two or more semen tests by the World Health Organization method is necessary to conclude efficacy, we believe that our supplement could be one treatment option for idiopathic male infertility, especially oligoasthenozoospermia, in the present situation in which there is no reliable treatment protocol.

## CONFLICT OF INTEREST

The authors declare no conflicts of interest in association with this manuscript.

## HUMAN RIGHTS STATEMENTS AND INFORMED CONSENT

This study was approved by the institutional review board of Juntendo University School of Medicine. All patients signed an informed written consent form before entering the study, and they were informed that they could terminate their cooperation with us whenever they wanted without any consequences.
